# Differentiated service delivery models among PLHIV in Akwa Ibom and Cross River States, Nigeria during the COVID‐19 pandemic: descriptive analysis of programmatic data

**DOI:** 10.1002/jia2.25820

**Published:** 2021-10-28

**Authors:** Olusola Sanwo, Navindra E. Persaud, Pius Nwaokoro, Augustine Idemudia, Uduak Akpan, Otoyo Toyo, Philip Imohi, Titilope Badru, Chika Obiora‐Okafo, Chimamaka Excellence Uzochukwu, Oluwapelumi Aliu, Kolawole Olatunbosun, Satish Raj Pandey, Hadiza Khamofu, Robert Chiegil, Ezekiel James, Isa Iyortim, Dorothy Oqua, Moses Bateganya

**Affiliations:** ^1^ FHI 360 Abuja Nigeria; ^2^ FHI 360 Washington DC USA; ^3^ AHNi Abuja Nigeria; ^4^ USAID Abuja Nigeria; ^5^ Howard University Global Initiative Abuja Nigeria

**Keywords:** differentiated service delivery, people living with HIV, treatment retention, viral suppression, COVID‐19, Nigeria

## Abstract

**Introduction:**

The rapid increase in the number of people living with HIV (PLHIV) on antiretroviral therapy (ART) in Akwa Ibom and Cross River states in Nigeria led to overcrowding at clinics. Patients were devolved to receive ART refills through five differentiated service delivery (DSD) models: fast‐track (FT), adolescent refill clubs (ARCs), community pharmacy ART refill programs (CPARPs), community ART refill clubs (CARCs) and community ART refill groups (CARGs) designed to meet the needs of different groups of PLHIV. In the context of COVID‐19‐related travel restrictions, out‐of‐facility models offered critical mechanisms for continuity of treatment. We compared retention and viral suppression among those devolved to DSD with those who continued standard care at facilities.

**Methods:**

A retrospective cohort study was conducted among patients devolved to DSD from January 2018 to December 2020. Bivariate analyses were conducted to assess differences in retention and viral suppression by socio‐demographic characteristics. Kaplan–Meier assessed retention at 3, 6, 9 and 12 months. Differences in proportions were compared using the chi‐square test; a *p*‐value of <0.05 was considered significant.

**Results:**

A total of 40,800 PLHIV from 84 facilities received ART through the five models: CARC (53%), FT (19.1%), ARC (12.1%), CPARP (10.4%) and CARG (5.4%). Retention rates at 6 months exceeded 96% for all models compared to 94% among those continuing standard care. Among those using DSD, retention rate at 12 months was higher among adults than children (97.8% vs. 96.7%, *p* = 0.04). No significant sex differences in retention rates were found among those enrolled in DSD. Viral suppression rates among PLHIV served through DSD were significantly higher among adults than children (95.4% vs. 89.2%; *p* <0.01). Among adults, 95.4% enrolled in DSD were virally suppressed compared to 91.8% of those in standard care (*p* <0.01). For children, 89.2% enrolled in DSD were virally suppressed compared to 83.2% in standard care (*p* <0.01).

**Conclusions:**

PLHIV receiving ART through DSD models had retention but higher viral suppression rates compared to those receiving standard care. Expanding DSD during COVID‐19 has helped ensure uninterrupted access to ART in Nigeria. Further scale‐up is warranted to decongest facilities and improve clinical outcomes.

## 1 INTRODUCTION

Globally, over 38 million people are living with HIV and 26 million are currently receiving lifelong antiretroviral therapy (ART) [1]. In 2016, the World Health Organization recommended a “test and treat” approach for all people newly diagnosed with HIV [2]. This recommendation was based on scientific evidence that early ART initiation reduces morbidity and mortality among people living with HIV (PLHIV) [3, 4, 5]. This expanded eligibility for treatment, while necessary to save lives, stretched already overburdened health systems in resource‐limited settings, such as Nigeria. To address this situation, complementary differentiated service delivery (DSD) models were introduced in addition to the routine hospital service delivery models in high‐burden countries. The DSD models implemented support the attainment of the global targets for HIV treatment while maintaining optimum quality of care [6] for PLHIV.

Although core principles of DSD are provision of client‐centred care and achieving health system efficiencies, variations in model implementation by location, settings, HIV population and individual client characteristics are expected [7, 8, 9]. In addition, for optimal outcomes, DSD models should be constantly adapted to address challenges of access, and quality of care and treatment outcomes for PLHIV [10]. Sub‐populations of PLHIV, such as pregnant and breastfeeding women, adolescents and children, men and key population members, may have different needs. Other individual characteristics of PLHIV accounted for during the design included clinical stage of disease and living environment. Across service characteristics (provider, location, frequency and intensity of care), different treatment delivery models are aimed at providing more client‐centric services [9].

Data from other studies suggest that DSD models for PLHIV are more resource efficient and do not compromise patient care [11, 12]. Uganda successfully implemented a DSD model using community drug distribution points for clients who were on ART for more than 3 months, showed good adherence (95%) and a CD4 count greater than 350 cells/mm^3^ [13]. Mozambique implemented patient‐managed community ART groups that led to significant improvement in ART retention and other treatment outcomes [10]. In South Africa, a high‐volume ART site provided multi‐month dispensing to stable clients through the fast‐track (FT) model resulting in significant reduction in client waiting time with better retention and satisfaction [3].

PLHIV in Nigeria, as in other countries in sub‐Saharan Africa, face significant challenges accessing ART [14, 15]. In Akwa Ibom (HIV prevalence 5.5%) and Cross River (HIV prevalence 2.2%) states, the high HIV burden and geographic access challenges further constrain access [16]. To close treatment gaps in these two states, the U.S. President's Emergency Plan for AIDS Relief (PEPFAR) through the United States Agency for International Development (USAID) funded the Strengthening Integrated Delivery of HIV/AIDS Services (SIDHAS) project to drive the surge implementation [17]. The surge response resulted in a marked increase in the number of PLHIV receiving ART in the two states. This increase in patient load was not accompanied by a corresponding increase in the number of healthcare workers (HCWs) at the public health facilities, which led to long wait times and overcrowding. To address this, starting in 2016, the project instituted DSD models to provide options for clients who wished to be devolved from the facilities. The rate of uptake of the devolvement options increased with the onset of COVID‐19 in February 2020.

The COVID‐19 outbreak in Nigeria was first reported on 27 February 2020, when the first confirmed case was announced by the Nigeria Center for Disease Control [18]. As of 17 February 2021, a total of 161,074 cases and 2018 deaths were reported by the Nigeria CDC. Several measures were instituted by the government, including total lockdowns in some states, restrictions on interstate movement, school closures, workplace restrictions and bans on social gatherings to help curb the spread of the virus. These restrictions prompted concerns about treatment interruption among PLHIV and necessitated a targeted intervention to encourage more patients to devolve from their usual treatment site to suitable DSD models to minimize the risk of exposure to COVID‐19 for HCWs and patients.

The objective of this study was to compare retention and viral load suppression among PLHIV in Akwa Ibom and Cross River states who received their ART refills through DSD models with those who continued to receive refills through standard care at facilities.

## 2 METHODS

### 2.1 SIDHAS project

The SIDHAS project supports the Government of Nigeria (GON) in implementing comprehensive HIV services in Akwa Ibom and Cross River states. The goal is to sustain cross‐sectional integration of HIV and AIDS services with tuberculosis (TB) services by building the capacity of GON staff to deliver high‐quality, comprehensive, preventive care and treatment and other related services. The project, which began in 2011, currently provides technical support to 151 health facilities (123 public, 26 private‐for‐profit and 2 faith‐based organizations) and 83 community pharmacies.

In the SIDHAS project, five DSD models were introduced to provide ART refills to the growing number of PLHIV on treatment. For the purpose of devolvement, stable clients were those who had been on ART for >12 months, had achieved at least 90% adherence, were VL suppressed (<1000 copies/ml) as at the time of the devolvement and had no opportunistic infections. The characteristics of the DSD models are summarized in Table 1. These models were designed to meet the unique needs of different groups and were introduced at different times.

**Table 1:** Description of models of HIV treatment

Building blocks of service delivery	Clinical consultations	ART refills	VL sample collection	Psychosocial support
Model	Standard of care			
Eligibility (who)	All patients are eligible			
When	Fixed working hours, normal wait time			
Location of services (where)	Health facility			
Fees	None			
Services provided	+	+	+	+
Model	Fast‐track			
Eligibility (who)	Only stable patients are eligible			
When	Fixed working hours, patients served within 5 min of arrival at facility			
Location of services (where)	Health facility			
Fees	None			
Services provided		+	+	+
Model	Adolescent refill clubs			
Eligibility (who)	Adolescents and young adults (10–24 years of age)			
When	Fixed after work hours on selected days			
Location of services (where)	Facility			
Fees	None			
Services provided		+	+	+
Model	Community pharmacy ART refill programs (CPARPs)			
Eligibility (who)	Stable adults (18 years and older)			
When	Flexible			
Location of services (where)	Private pharmacies in the community			
Fees	Yes			
Services provided		+		+
Model	Community ART refill groups (CARGs)			
Eligibility (who)	All patients linked through family or group membership			
When	Flexible hours			
Location of services (where)	Client's homes			
Fees	None			
Services provided		+		+
Model	Community ART refill clubs (CARCs)			
Eligibility (who)	All patients			
When	Flexible			
Location of services (where)	Convenient community locations, that is clinics and schools			
Fees	None			
Services provided	+	+	+	+

ART, antiretroviral therapy; VL, viral load.

### 2.2 Data collection

For this study, de‐identified data were extracted from Lafiya Management Information System (LAMIS), an electronic medical record database, that houses routine programmatic data collected from PLHIV who access services at SIDHAS‐supported health facilities. These service delivery data are collected using standardized paper‐based forms at each patient encounter and then entered into LAMIS by facility staff. The database was reviewed, and all PLHIV who were enrolled in one of the DSD models up to 30 December 2020 were selected for inclusion in the study. Data extracted for each patient included basic demographic information: age and sex; and clinical information: DSD models to which they were devolved, date devolved and recent viral load test results at the time of the study. The extracted data contained no patient names or any other personal identifying information that could be used to identify individual patients. The extracted data were subjected to internal consistency checks and assessed for outliers, which were removed prior to analysis.

### 2.3 Data quality measures

At the end of each day, patient data initially captured on paper are entered into LAMIS by data entry clerks attached to each clinic. The data were summarized at the end of each week showing the number of individuals who accessed different services. All data were validated internally on a regular basis following established processes for data quality assurance setup by the SIDHAS project. Summary reports submitted to the project were compared with source documents, such as registers and other intake forms in the facilities to ensure consistency. If discrepancies were observed in the data, then reasons for the discrepancies were ascertained, noted and the data in LAMIS were adjusted to ensure consistency with the source document.

### 2.4 Data analysis

Individuals were considered to still be in care if their next pickup date for ART from their designated pickup point (for the DSD group) or the health facility (for the non‐DSD group) was after 31 December 2020. Individuals were classified as virally suppressed if their viral load was <1000 copies/ml.

Time‐based cohorts of patients devolved to the DSD models were created based on the simplified cohort analysis approach, commonly used during routine ART program monitoring [19]. With this approach, patients were placed in different cohorts based on the dates on which they were enrolled in one of the DSD models. Patients devolved during any given quarter (3‐month period) were considered to be in the same cohort.

Descriptive statistics were used for characteristics of PLHIV who were enrolled in DSD models. Bivariate analyses were then conducted to assess differences in retention and viral suppression rates by socio‐demographic characteristics. Kaplan–Meier was used to assess retention for up to 12 months for those individuals who were enrolled in the DSD models. The Log‐rank test was used to assess differences in retention rates by age and sex across the DSD models. Differences in proportions of individuals who were virally suppressed across the DSD and non‐DSD models were compared using chi‐square test. All tests were considered significant with a *p*‐value of < 0.05.

### 2.5 Ethical consideration

This study was reviewed by the Protection of Human Subjects Committee at FHI 360 and was categorized as non‐human subject research. The data for this study were collected from an existing project database that is used for routine patient management and program monitoring. The authors had no access to the patients or any personal identifying information for the individuals who were included in the study.

## 3 RESULTS

### 3.1 Patients and models

At the end of December 2020, a total of 133,644 PLHIV were receiving ART at SIDHAS‐supported facilities in Akwa Ibom and Cross River states. Out of those, 40,800 (30.5%) had been devolved to receive ART refills through five DSD models, and 92,844 (69.5%) continued to receive ART at the facilities where they were enrolled. The rate of devolution started slowly but then increased significantly after June 2020 during the first wave of the epidemic in Nigeria (Table 2).

**Table 2:** Number of PLHIV devolved at different times

Time period	Number (%) devolved
January 2018–December 2019	3250 (7.96%)
January–March 2020	3821 (9.4%)
April–June 2020	3359 (8.2%)
July–September 2020	12,528 (30.7%)
October–December 2020	17,842 (43.7%)
Total	40,800 (100%)

PLHIV, people living with HIV.

Most patients were devolved to the community ART refill club (CARC) model (Table 3). PLHIV less than 20 years old were significantly more likely than those older than 20 to have been devolved to one of the DSD models; 42% (2912/6904) of those less than 20 years old were devolved compared to 29.8% (37,888/126,904) of those 20 or older (*p* < 0.05). There were no significant differences in the proportion of males and females devolved to one of the DSD models.

**Table 3:** Characteristics of people receiving treatment through different methods

	CARC *n* (%)	FT *n* (%)	ARC *n* (%)	CPARP *n* (%)	CARGs *n* (%)	Standard care *n* (%)
Sex						
Male	8525 (39.6)	2208 (28.4)	979 (19.19)	1534 (36.2)	828 (37.9)	33,047 (35.5)
Female	13,000 (60.4)	5569 (71.6)	3933 (80.1)	2705 (63.8)	1355 (62.1)	59,961 (64.5)
Age (years)						
<20	721 (3.3)	193 (2.5)	1829 (37.1)	38 (0.9)	131 (6.0)	3992 (4.3)
≥20	20,867 (96.7)	7591 (97.5)	3098 (62.9)	4278 (99.1)	2054 (94.0)	89,016 (95.7)
Median (IQR)	35 (29–42)	37 (31–45)	20 (18–22)	41 (35–48)	34 (28–41)	36 (29–43)
Total	21,588	7784	4927	4316	2185	93,008

Abbreviations: ARC, adolescent refill clubs; CARC, community ART refill clubs; CARG, community ART refill groups; CPARP, community pharmacy refill programs; FT, fast track; IQR, inter‐quartile range.

### 3.2 Viral suppression

Overall viral suppression was higher among DSD participants compared to those who continued to receive standard care at facilities (94.9% vs. 91.5%; *p* < 0.05). Among patients on DSD, viral load suppression rate was highest among those devolved to the FT model (98%) and lowest for those assessing care through the adolescent refill club (ARC) (90%) (Table 4).

**Table 4:** Viral suppression rates for patients disaggregated by model of care

	Standard care	DSD model *N* = 40,800				
	*N* = 93,008	ARC	CARC	CPARP	FT	CARG
Number who had VL test	63,093	3816	15,023	3455	7227	1793
Number suppressed	57,705	3420	14,185	3310	7089	1717
% suppressed	91%	90%	94%	96%	98%	96%

Abbreviations: ARC, adolescent refill clubs; CARC, community ART refill clubs; CARG, community ART refill groups; CPARP, community pharmacy refill programs; DSD, differentiated service delivery; FT, fast track; VL, viral load.

Viral suppression rates were consistently higher among persons on DSD compared to those receiving the standard care (Table 5). Among persons 20 years or older, 95.4% of those enrolled in DSD were virally suppressed compared to 91.8% receiving standard care (*p* < 0.01). Similarly, for those younger than 20 years, 89.2% enrolled in DSD were virally suppressed compared to 83.2% who received ART at clinics (*p* < 0.01). Among females, 94.7% of those enrolled in DSD were virally suppressed compared to 91.7% receiving standard care (*p* < 0.001). A higher proportion of males enrolled in DSD (95.3%) were virally suppressed compared to males receiving standard care (90.9%) (*p* < 0.001).

**Table 5:** Viral suppression rates disaggregated by models of care and age group

Demographic characteristics	Standard care versus DSD	% virally suppressed	Number tested	*p*‐value
Age				
< 20 years	Standard care	83.2	2889	<0.001
	DSD	89.2	2377	
20 + years	Standard care	91.8	60,364	<0.001
	DSD	95.4	28,937	
Sex				
Male	Standard care	90.9	21,254	<0.001
	DSD	95.3	10,496	
Female	Standard care	91.7	41,999	<0.001
	DSD	94.7	20,818	

Abbreviation: DSD, differentiated service delivery.

### 3.3 Retention in care

**Figure 1:**
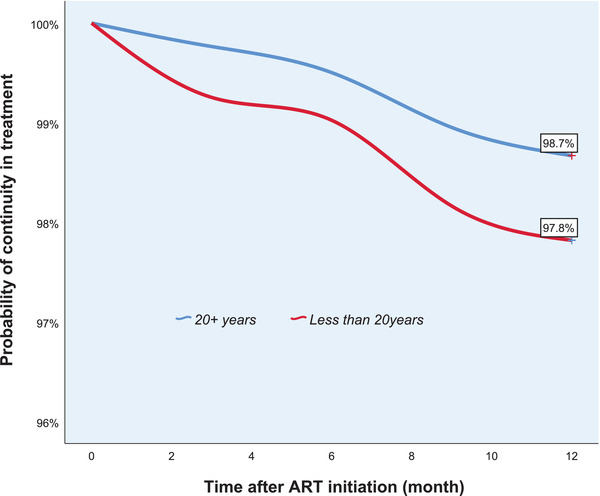
Twelve‐month retention among patients on differentiated service delivery. ART, antiretroviral therapy.

Among those who were devolved to DSD (Figure 1), retention rates at 12 months were significantly higher among those who were 20 years or older compared to those less than 20 years (*p* = 0.004). No significant differences in 12‐month retention rates were found between males and females (*p* = 0.592).

Table 6 summarizes retention among PLHIV based on the simplified cohort analysis approach. With this analysis, we found that retention rates drop off as cohorts “age”. Among the cohort followed up for 3 months, retention was 99.5%; in the 6‐month cohort, 98.4%; in the 9‐month cohort, 97.0%; and for those in the 12‐month cohort, retention dropped to 89.5%.

**Table 6:** Retention rates for patients disaggregated by model of care

Retention by aggregated period – total DSD								
Period	Elements	ARC	CARC	CPARP	Fast track	F‐CARG	S‐CARG	Total
3 months (July–Sept 2020)	Number devolved	1921	7385	237	2266	592	171	12,572
	Number continued in treatment	1912	7347	235	2260	590	171	12,515
	% continued in treatment	99.5%	99.5%	99.2%	99.7%	99.7%	100.0%	99.5%
6 months (April–June 2020)	Number devolved	340	1805	739	404	82	28	3398
	Number continued in treatment	332	1775	728	400	82	28	3345
	% continued in treatment	97.6%	98.3%	98.5%	99.0%	100.0%	100.0%	98.4%
9 months (Jan–March 2020)	Number devolved	202	2637	476	270	326	0	3911
	Number continued in treatment	196	2540	465	270	322	0	3793
	% continued in treatment	97.0%	96.3%	97.7%	100.0%	98.8%	0%	97.0%
12 months (Oct–Dec 2019)	Number devolved	58	382	111	7	3	3	564
	Number continued in treatment	56	330	109	7	0	3	505
	% continued in treatment	96.6%	86.4%	98.2%	100.0%	0.0%	100.0%	89.5%

ARC, adolescent refill clubs; CARC, community ART refill clubs; CARG, community ART refill groups; CPARP, community pharmacy refill programs; DSD, differentiated service delivery.

## 4 DISCUSSION

In this paper, we describe DSD models and compare viral suppression and retention among PLHIV who were devolved to receiving care through various DSD models with those who continued in standard, facility‐based care in two states in Nigeria. Close to one‐third of patients (30.3%) were devolved to receive care through the five DSD models. Enrolment of patients into the different models increased over the 2‐year period from January 2018 to December 2020 with the most significant increase occurring in July 2020, which coincided with the peak of the first wave of the COVID‐19 pandemic in Nigeria. The movement restrictions, physical distancing requirements, supply chain disruptions and financial difficulties brought on by the pandemic necessitated the transitioning of patients to other models of care that limit exposure of both patients and HCWs to COVID‐19 [20]. The results of our study are consistent with others that have reported some clients are very amenable to receiving care and treatment out of the healthcare facility [21]. To inform scale up, it is important to continually review routinely collected data to understand how treatment outcomes in DSD models compared with the standard of care.

Overall, we found higher suppression but similar retention rates among patients enrolled in the DSD models compared to those who continued to receive services through standard care at the facilities. Viral suppression rates for patients devolved to the DSD models were 95% compared to 91% among those who continued to receive standard care. Among models, viral suppression rates were highest with FT and lowest in the ARCs. The DSD models offer options for patients without compromising quality of care [22]. These models need to be continuously evaluated to ensure that they meet client needs and assure quality. The experience managing patients who were devolved before the COVID‐19 outbreak helped to catalyze the rates at which patients were devolved and to maintain the quality of service. In the COVID‐19 context, engagement with stakeholders is critical to avoid suboptimal outcomes [20]. The SIDHAS team offered clients a number of models in the two states to cater to the unique needs of clients. The CPARP and CARC models are critical for optimizing healthcare services, especially for patients living in remote areas with bad road networks and poor coverage of health facilities. Patients in these models are supported by HCWs who directly ensure they receive the same comprehensive healthcare package as provided at a health facility.

The cost of accessing treatment is a major factor affecting continued access to ART among patients on treatment [23]. While DSD models offer greater flexibility, out of facility models can, however, be more expensive than conventional facility care for equal or improved outcomes [24]. Donors and program managers would need to take this into account when planning and scaling up DSD. Retention rates among patients in the fee‐paying CPARP model were 98.2%, which was marginally lower than those who continued to use the FT model care at the facilities for free. The CPARP model still offers an opportunity for busy patients in urban settings who are able to pay a small user fee. In other studies, user fees have had a mixed impact on access to services, especially in West Africa [25]. During the COVID‐19 pandemic when movement was restricted and the cost of transportation increased, we observed increased enrolment in this DSD program.

Although DSD models were associated with high retention, implementing them in the middle of the COVID‐19 pandemic would need some adjustments to ensure they meet the preferences of the patients to ensure optimal utilization [26]. The number of patients on antiretrovirals who chose different DSD models has implications for programming. The majority (78.6%) of patients in our project who were eligible for DSD continue to receive facility‐based care either through standard care, FT or ARCs. The FT model, which requires patients to go to the health facility, nevertheless, ensures that the waiting time is reduced to the barest minimum. Reducing the waiting time helps improve treatment outcomes and may also act as a motivation to unstable clients who are assessing care at the health facility [22]. Retention was highest with the FT model highlighting its potential for patients who prefer facility models. Other authors have shown that some patients find it easier to access medication at facilities [27]. As multi‐month dispensing, especially for 6‐month supplies, scales up, the FT model holds promise. Waiting time in this model could be further reduced through introduction of automated lockers and prefabricated pharmacy in a box conveniently placed in less busy parts of a health facility. With this, patients on FT can pick up their medication without having to register when they visit the clinic.

We found higher retention (98.2%) among children in the ARCs than their peers who continued to receive standard care at facilities (93.6%). This model, which offers adolescents a platform to relate and interact with their peers, gives them a sense of belonging and hope that may help address the viral suppression gaps among adolescents.

Our study had some limitations. We used programmatic data for this analysis and as such, there are a number of limitations. Firstly is the inherent selection bias as participants were not randomized to the respective DSD models but elected to join them when they were offered. Secondly, the eligibility criteria for the DSD models required clients to be stable on treatment. These clients would more likely also be retained in care and maintain their VL suppression. Thirdly, the majority of the patients were devolved during the last 6 months, resulting in a relatively short follow‐up period resulting in limited ability to make inferences about the longer term outcome across the DSD models. Finally, these data were not collected for research purposes and may contain some level of errors, including missing data and inconsistencies, that could affect generalizability of the results. Finally, data for other important confounding variables that could have affected the relationships were not collected and the relationships could not be adjusted for these. Consistent data quality assurance measures implemented by the project, including regular review of the data collection tools and mentoring of staff, helped mitigate this situation.

## 5 CONCLUSIONS

PLHIV receiving ART through DSD models had better treatment retention and viral suppression rates than those receiving ART through standard care at facilities. Expanding DSD treatment models during the COVID‐19 pandemic has helped ensure uninterrupted access to ART in Nigeria. Further scale‐up of various DSD models is warranted to decongest facilities and improve clinical outcomes among PLHIV. These data, collected during routine program implementation, represent the real‐world setting and provide an example of routinely collected data can be used to answer important research questions. Persons working in other settings who are thinking of adapting these models should use their data to adjust them to suit their unique context [28].

## COMPETING INTERESTS

The authors report no competing interests.

## AUTHORS’ CONTRIBUTIONS

OS, NP, PN, AI and MB conceptualized the study and supervised the analysis and interpretation of the data. CU, OA, PI and CO organized and prepared the data for analysis. AI, UA, OT and TB conducted the data analysis with the advice of OS, NP and PN. OS, NP, PN and AI wrote the first draft of the manuscript. KO, HK, SP, RC, EJ, IL and DO reviewed the draft and added content to specific sections. All authors contributed to data interpretation and approved the final version.

## FUNDING

This publication resulted in part from data collected during implementation of the PEPFAR‐funded SIDHAS project in Nigeria (Cooperative Agreement Number: AID‐620‐A‐11‐00002).

## DISCLAIMER

The content of this article represents the views of the authors and does not necessarily represent the views of the funder.
